# Partizipation in der Versorgungsforschung: Die Entwicklung eines vorläufigen Frameworks für gemeinsames Forschen mit Menschen mit Demenz, Angehörigen und verschiedenen Berufsgruppen

**DOI:** 10.1007/s00391-025-02494-6

**Published:** 2025-09-06

**Authors:** Anna Louisa Hoffmann-Hoffrichter, Christina Manietta, Mike Rommerskirch-Manietta, Martina Roes

**Affiliations:** 1 Deutsches Zentrum für Neurodegenerative Erkrankungen e. V., Stockumer Str. 12, 58453 Witten, Deutschland; 2 Witten/Herdecke University (UW/H), Faculty of Health, Department of Nursing Science, Alfred-Herrhausen-Straße 50, 58448 Witten, Deutschland

**Keywords:** Partizipative Forschung, Versorgungsforschung, Menschen mit Demenz, Framework, Participatory research, Health services research, People with dementia, Framework

## Abstract

**Hintergrund:**

Modelle, die Menschen mit Demenz und Angehörige sowie Expert:innen aus verschiedenen Versorgungssettings in Forschung aktiv einbeziehen, sind bisher im deutschsprachigen Kontext nicht implementiert. Diese sind notwendig, um auch die Perspektive der „Mitforschenden“ aktiv einzubinden und Themen aufzugreifen, die sich am Alltag von Menschen mit Demenz und ihren Angehörigen und in der Versorgungspraxis Tätigen orientieren.

**Ziel der Arbeit:**

Das Ziel ist die Entwicklung eines vorläufigen Frameworks für gemeinsames Forschen.

**Material und Methoden:**

Das Vorgehen beinhaltet einen partizipativen Ansatz mit Stakeholdern verschiedener Personengruppen. Dazu zählen Menschen mit Demenz, Angehörige sowie Expert:innen aus dem langzeit- und akutstationären Setting. Es wurde ein Modell der partizipativen Forschung in die deutsche Sprache übersetzt und in virtuellen Meetings mit Stakeholdern besprochen, diskutiert und konsentiert. Die Ergebnisse wurden in einem Workshop besprochen, um die als notwendig erachtete Unterstützung der „Mitforschenden“ herauszuarbeiten. Die Treffen wurden in Protokollen festgehalten und inhaltsanalytisch deduktiv induktiv ausgewertet.

**Ergebnisse:**

Insgesamt nahmen 13 Stakeholder an dem partizipativen Vorhaben teil. Das Framework für gemeinsames Forschen beinhaltet Grundprinzipien, Tätigkeiten und Unterstützungsbedarfe des gemeinsamen Forschens.

**Diskussion:**

Neben Expert:innen aus unterschiedlichen Settings der Gesundheitsversorgung möchten und können auch Menschen mit Demenz und Angehörige aktiv in Forschung einbezogen werden. Um das vorläufige Framework weiterzuentwickeln, wird es in Projekten partizipativer Forschung am Deutschen Zentrum für Neurodegenerative Erkrankungen (DZNE) Standort Witten erprobt und verdichtet.

**Zusatzmaterial online:**

Zusätzliche Informationen sind in der Online-Version dieses Artikels (10.1007/s00391-025-02494-6) enthalten.

## Abstract (en)

**Background:**

Models that actively involve people living with dementia and their relatives as well as experts from various healthcare settings in research have not yet been implemented in the context of health services research in the German language. Such models are necessary to actively involve the perspectives of “co-researchers” and address topics pertaining to the everyday lives of people living with dementia and their relatives as well as individuals working in healthcare practice.

**Objective:**

The aim of this study is to develop a preliminary framework for joint research.

**Material and methods:**

The procedure used in this research features a participatory approach involving stakeholders recruited from different groups of people, including people living with dementia, their relatives and experts from long-term and acute inpatient settings. A participatory research model was translated into German and discussed, debated and agreed upon in virtual meetings with the stakeholders. The results were discussed in a workshop with the goal of determining the types of support that were considered necessary for “co-researchers”. All meetings were documented in protocols that were analyzed both deductively and inductively.

**Results:**

A total of 13 stakeholders participated in this project. The preliminary framework for joint research contains basic principles, activities and support requirements for joint research.

**Conclusion:**

In addition to experts from various healthcare settings, people living with dementia and their relatives both want to and can be actively involved in research. To refine the preliminary framework, it continues to be tested and developed in the context of participatory research projects at the German Center for Neurodegenerative Diseases (DZNE) site Witten.

## Einleitung

Im deutschsprachigen Raum entwickelte sich partizipative Forschung sukzessive in den letzten 25 Jahren, auch durch die Gründung des Netzwerks für Partizipative Gesundheitsforschung [8]. In einigen Ländern (z. B. United Kingdom) ist partizipative Forschung ein methodisches Vorgehen, das in der Versorgungsforschung etabliert ist [2]. Menschen mit Demenz werden selten aktiv in Versorgungsforschung in Deutschland eingebunden [7]. Um Forschende in der Umsetzung zu unterstützen, bedarf es eines Modells, das Prinzipien und Tätigkeitsbereiche festhält.

## Hintergrund und Fragestellung

Der Einbezug von Personen der Öffentlichkeit in Gesundheits- und Versorgungsforschung wird als partizipative Gesundheitsforschung bezeichnet, um die Beteiligung dieser Personen in allen Schritten des Forschungsprozesses zu steigern [10]. Zu diesen Personengruppen zählen Menschen mit einer Erkrankung, deren An‑/Zugehörige sowie Personen der Gesundheitsprofessionen und -versorgung. Das Ziel ist es, wissenschaftlich hierarchische Strukturen zu dezentralisieren [20] sowie beteiligte Akteur:innen individuell und kollektiv zu ermächtigen [19]. Das kann die Relevanz des Forschungsgegenstandes für die Personengruppen verbessern. Gleichzeitig ermöglicht der Einbezug von Personen der Öffentlichkeit nicht nur einen größeren Personenkreis, der an der Forschung mitwirkt, sondern auch verbesserte Möglichkeiten der Gewinnung heterogener Populationsgruppen sowie die Dissemination von generiertem Wissen über die wissenschaftliche Community hinaus [6]. Der Einbezug dieser Personengruppen erhöht das Vertrauen der Endnutzenden in die Forschungsergebnisse [9]. Herausforderungen sind z. B. Konflikte unter Personengruppen aufgrund heterogener Expertise, Prioritäten und Erwartungen, die kontinuierliche Reflexion und Selbstreflexion oder die Angemessenheit forschungsethischer Grundsätze [19].

International gibt es Handlungsrahmen zum Einbezug unterschiedlicher Personengruppen. Die Einbindung umfasst Tätigkeitsbereiche, wie die Rekrutierung, die aktive Einbindung als Mitforschende in Datenerhebung, -auswertung oder Dissemination [12]. Es werden verschiedene Ausprägungen der Einbindung [2], die von geringem bis hohem Ausmaß reichen, unterschieden. Solche Modelle ermöglichen Beteiligten, eine eigenständige Beurteilung und Reflexion der jeweiligen Einbindung vorzunehmen [21]. Aner [1] beschreibt Kriterien, die in der Durchführung partizipativer Methoden umgesetzt werden sollen, wie u. a. die Reflexion des Kontextes, die Abgabe von Macht an die Beteiligten, die Transparenz unterschiedlicher Ausgangspunkte sowie die gemeinsame Planung und Durchführung von Partizipation [1].

International ist in der Versorgungsforschung von Menschen mit Demenz der aktive Einbezug dieser Personengruppe nicht wegzudenken, um zu Relevanz, Akzeptanz, Ethik und Qualität von Forschung [5, 7] sowie zur Zufriedenheit der Verbraucher:innen [1] beizutragen. In Deutschland ist die Partizipation von Menschen mit Demenz noch nicht weit fortgeschritten [3], obwohl sie Verbrauchenden die Möglichkeit bietet, auf die eigenen Lebensverhältnisse Einfluss zu nehmen [1]. Um dies zu fördern, braucht es universelle Grundsätze der Beteiligung in der Forschung von Menschen mit Demenz [13] und damit ein Bewusstsein der Forschenden für Demenz [14]. Modelle, die Menschen mit Demenz einbeziehen und deren Unterstützungsbedarf berücksichtigen, können deren Einbezug in Forschung unterstützen, indem sie Prinzipien und Möglichkeiten sowie zu beachtende Aspekte des Einbezugs adressieren. Nur durch den Einbezug der Perspektive von Menschen mit Demenz und ihren Angehörigen kann Forschung zu dieser Zielgruppe die für sie relevanten Themen adressieren und die Relevanz der Forschung sowie die Durchführung des Forschungsprozesses überhaupt erst begründen [7]. Dabei ist es im Kontext der „echten“ Partizipation [1] von besonderer Relevanz, soziale Inklusion und Chancengleichheit mitzudenken und umzusetzen [1]. Griffith et al. [7] halten Empfehlungen zum Einbezug von Menschen mit Demenz und ihren Angehörigen in einem Best-Practice Rahmenwerk vor. Sie beschreiben die Relevanz wiederkehrender, flexibler Treffen zum Beziehungs- und Vertrauensaufbau, zur Festlegung gruppenspezifischer Kommunikationsstrategien, des Einklangs von Forschungsgegenstand mit gelebten Erfahrungen der Menschen mit Demenz, des Einbezugs von Menschen mit Demenz im gesamten Forschungsprozess sowie eines qualifizierten Vermittlers [7].

Modelle, die den aktiven Einbezug in Forschung von Menschen mit Demenz und Angehörigen zusammen mit Expert:innen aus unterschiedlichen Versorgungssettings fokussieren, sind für den deutschsprachigen Kontext zum jetzigen Zeitpunkt nicht zu identifizieren.

Das Ziel war es, ein erstes vorläufiges Framework für gemeinsames Forschen zwischen Menschen mit Demenz, Angehörigen sowie Mitarbeitenden einer Forschungsinstitution und Expert:innen aus der langzeitstationären Altenhilfe und dem akutstationären Setting zu erstellen. Daraus leiten sich folgende Fragen ab:

1. Welche Ausprägungen der Einbindung können sich die verschiedenen Personengruppen für eine Zusammenarbeit in der Versorgungsforschung vorstellen?
2. Welche Konsequenzen ergeben sich hieraus für das gemeinsame Forschen, sowohl für die professionellen Forschenden als auch die Mitforschenden?
3. Welche Unterstützung benötigen die verschiedenen Personengruppen?

## Methode

Das Vorgehen beinhaltet die Übersetzung und kulturelle Adaption des Modells von Finderup et al. [4] durch die Autor:innen sowie die Besprechung dieses überarbeiteten Modells im Rahmen von Meetings und einem Workshop mit Stakeholdern. Das Vorgehen der Meetings und des Workshops orientiert sich an einem partizipativen Ansatz, bei dem die Teilnehmenden das überarbeitete Modell diskutierten, reflektierten und mitgestalteten. Nach dem Stufenmodell der Partizipation entspricht diese Vorgehensweise bereits der Stufe der Partizipation [21]. Kriterien [1], die an dieses Vorgehen angelegt wurden, sind, dass die Meetings und der Workshop in einen größeren Kontext mit verschiedenen Stakeholdern eingebunden wurden, die Stakeholder die Benennung der Zusammenarbeitsformen mitbestimmen konnten, die Ausgangspositionen der Stakeholder und Forschenden transparent gemacht wurden und damit die Lernprozesse in partizipativen Methoden aller Beteiligen gefördert wurden.

Methodische Erläuterungen zum theoretischen Rahmen, zur Rekrutierung der Stakeholder, zur Ethik sowie nähere Erläuterungen zu den Stakeholdern können dem Zusatzmaterial online entnommen werden.

### Stakeholder/Sample

Es haben 13 Stakeholder an den Meetings teilgenommen. Hierzu zählen 2 Menschen mit Demenz, 2 Angehörige, 5 Expert:innen aus der Altenhilfe sowie 4 Expert:innen aus dem akutstationären Setting. Von den Stakeholdern haben 12 an den virtuellen Meetings und 9 Personen an dem Workshop teilgenommen. Gründe für eine Nichtteilnahme an dem Workshop waren mangelnde zeitliche Ressourcen.

### Meetings mit den Stakeholdern

#### Setting

Da sich die Stakeholder in verschiedenen geografischen Regionen zu den Forschenden befanden, fanden die Meetings virtuell über die Videokommunikationsplattform Zoom statt. Der Workshop wurde in einer barrierefreien Räumlichkeit in Nordrhein-Westfalen durchgeführt. Die Meetings wurden homogen mit jeweiligen Personengruppen durchgeführt.

#### Vorbereitung der Meetings

Es wurden Präsentationen in einfacher Sprache vorbereitet, um niederschwellig Informationen zum Modell nach Finderup et al. [4] vorzustellen. Die Unterlagen erhielten die Teilnehmenden vor dem Treffen per E‑Mail.

#### Ablauf der Meetings

Die virtuellen Meetings wurden von Oktober bis November 2022 durchgeführt. Nach einer Vorstellungsrunde und einer Vorstellung zu dem Modell der 5 Ausprägungen der Einbindung in Forschung fand im Anschluss ein Austausch statt. Hier erläutertem Stakeholder ihre Perspektive hinsichtlich der Ausprägung, möglicher Formen der Zusammenarbeit innerhalb der Personengruppen und sich daraus ableitender notwendiger Unterstützungsbedarfe. Die Meetings wurden schriftlich in Ergebnisprotokollen festgehalten.

### Workshop

#### Ablauf, Setting

Mit Abschluss des Projektvorhabens wurde am 08.12.2022 ein Workshop in Präsenz durchgeführt, zu dem alle Stakeholder aus den virtuellen Meetings eingeladen wurden.

#### Vorbereitung des Workshops

Die Ergebnisse der Meetings wurden durch ALH und MR zusammengefasst und in einer Präsentation für die Abschlussveranstaltung festgehalten.

#### Ablauf des Workshops

**Abb. 1:**
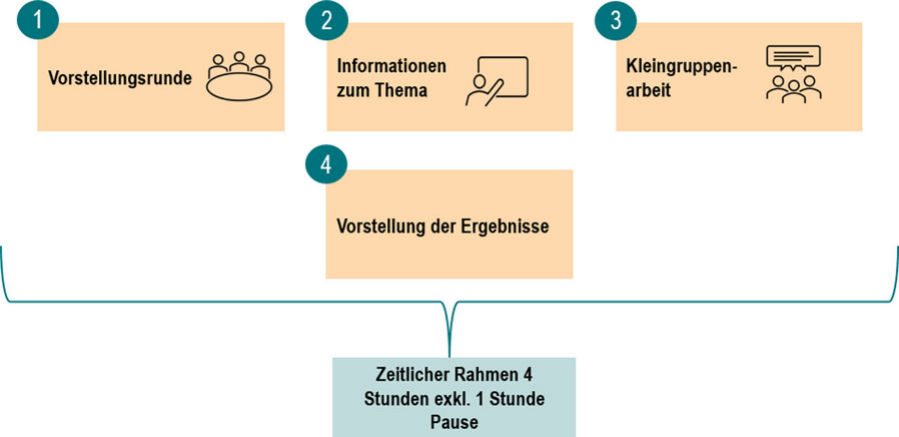
Ablauf des Workshops

Der Workshop (Abb. 1) beinhaltete eine Vorstellungsrunde und die Ergebnisvorstellung aus den Meetings. Anschließend erfolgte eine 60-minütige Kleingruppenarbeit der Stakeholder. Die Kleingruppen wurden so zusammengesetzt, dass je 2 Stakeholder unterschiedlicher Personengruppen die Aufgabenstellung bearbeiteten. Dabei wurden sie von den Forschenden unterstützt. Das Ergebnis umfasste die schriftliche Darstellung ihrer Vorstellungen zur Zusammenarbeit sowie der hierzu benötigten Unterstützung mit anderen Personengruppen auf einer Metaplantafel. Ihre Ausarbeitungen wurden im Plenum präsentiert. Der Workshop wurde protokolliert; die Metaplantafeln wurden fotografisch dokumentiert.

#### Datenanalyse

Die Protokolle und Fotos der Metaplantafeln wurden in MAXQDA (VERBI Software, Berlin, Deutschland) [18] überführt und mittels inhaltlich strukturierender qualitativen Inhaltsanalyse nach Kuckartz und Rädiker [11] deduktiv induktiv ausgewertet.

## Ergebnisse

### Gemeinsames Forschen und benötigte Unterstützung

**Abb. 2:**
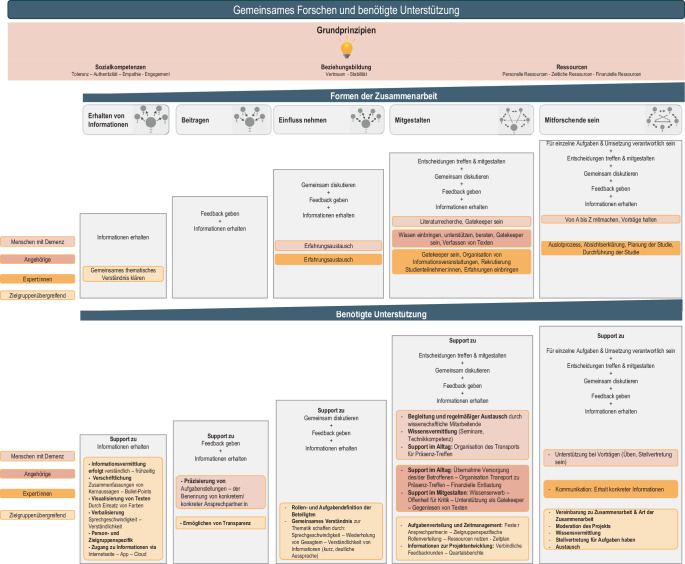
Ergebnistabelle

Im Folgenden werden die Hauptkategorien „Grundprinzipien der Zusammenarbeit“, „Formen der Zusammenarbeit“ und „Benötigte Unterstützung“ beschrieben (Abb. 2). Die Formen der Zusammenarbeit überschneiden und ergänzen sich, auch wenn sie getrennt voneinander dargestellt werden.

### Grundprinzipien der Zusammenarbeit

Die Personengruppen nannten Voraussetzungen, um gemeinsam zu forschen. Je mehr Voraussetzungen vorhanden sind, desto umfangreicher können sie in ein Forschungsprojekt eingebunden werden. Menschen mit Demenz und ihre Angehörige sehen Sozialkompetenzen anderer Personengruppen und den Aspekt der Beziehungsbildung als notwendige Faktoren an. Expert:innen sehen personelle, zeitliche als auch finanzielle Ressourcen als Kriterien an, ohne die sie nicht an gemeinsamen Forschungsprojekten mitwirken können. Häufig wird anhand dieser Kriterien entschieden, ob und in welchem Umfang sie eine Einbindung für sich ein- oder ausschließen.

### Formen der Zusammenarbeit

1. Zusammen mit den Stakeholdern wurden die Formen der Zusammenarbeit nach Finderup et al. [4] bestätigt und wie folgt benannt: I „Erhalten von Informationen“, II „Beitragen“, III „Einfluss nehmen“, IV „Mitgestalten“ und V „Mitforschende sein“. Die favorisierten Formen der Zusammenarbeit und Einbindung werden durch die Stakeholder auf dem Kontinuum von passiv zu aktiv vermehrt beschrieben:
2. *„Erhalten von Informationen“*: Der zielgruppenübergreifende Fokus liegt darin, ein thematisches Verständnis des Projektes zu entwickeln.
3. *„Beitragen“*: Es wurden keine weiteren Konkretisierungen beschrieben.
4. *„Einfluss nehmen“*: Menschen mit Demenz und Expert:innen beider Settings nannten den Erfahrungsaustausch. Dieser beinhaltet sowohl das Einbringen eigener Vorstellungen und Erfahrungen sowie die Diskussion zum Forschungsgegenstand oder zu Entscheidungen im Forschungsprozess.
5. *„Mitgestalten“*: Wurde als die häufigste Art der Zusammenarbeit mit Forschenden beschrieben. In Bezug auf die Rekrutierung von Personen, die zu einem Thema Feedback geben und gemeinsam diskutieren, sehen sich Menschen mit Demenz auch als Gatekeeper zu Selbsthilfegruppen für Menschen mit Demenz oder zu (oft geschlossenen) Gruppen in Sozialmedien, denen sie angehören. Menschen mit Demenz sehen sich auch in der Durchführung von Literaturrecherchen. Angehörige sehen sich daneben beispielsweise in einer Unterstützungs- und Beratungsfunktion durch Einbringen ihrer beruflichen Expertise (beispielsweise als gesetzliche Betreuung). Auch die Unterstützung und das Verfassen von Texten wurden benannt. Expert:innen sehen sich neben der Funktion des Gatekeepers zu beispielsweise Studierenden auch in der Organisation von Informationsveranstaltungen, der Rekrutierung von Studienteilnehmenden und das Einbringen ihrer persönlichen Erfahrungen.
6. *„Mitforschende sein“*: Für Menschen mit Demenz beinhaltet „Mitforschende sein“, dass sie in allen Ausprägungen Tätigkeiten übernehmen und ausführen. Für Expert:innen bedeutet es, Verantwortung zu übernehmen. Zu Beginn der jeweiligen Studie wird der Eigenverantwortung ein Auslotprozess zusammen mit der Gruppe vorangestellt. Es wird abgewogen, welche konkreten Tätigkeiten im Forschungsprojekt eigenverantwortlich durchgeführt werden können. Dieses Handeln wird auch durch die persönliche Erfahrung mit vorherigen Forschungsprojekten beeinflusst. Eigenverantwortliche Tätigkeitsfelder beinhalten für sie, die Studie zu planen und durchzuführen.

### Benötigte Unterstützung

Die Personengruppen benötigten Unterstützung, um gemeinsam zu forschen. Diese Notwendigkeit nimmt mit der Ausprägung der Zusammenarbeit zu, da sie die Unterstützung der unteren Ausprägungen miteinschließt.

#### I „Erhalten von Informationen“.

Für ein gemeinsames thematisches Verständnis bedarf es verschiedener Arten von Informationsvermittlung. Menschen mit Demenz benötigen verschriftlichte Informationen, verständlich, in kurzen Zusammenfassungen von Kernaussagen aufbereitet, in einfacher Sprache und der Nutzung von Bullet Points. Die Visualisierung von Informationen wird durch Farbeinsatz erleichtert. Die Verbalisierung von Informationen wünschen sich Menschen mit Demenz auf Augenhöhe in moderater Sprechgeschwindigkeit unter Nutzung verständlicher Begriffe, die sich in verschriftlichten Informationen wiederfinden. Die Personengruppen benötigten Unterstützung in der Art der Informationsvermittlung, die angepasst an beispielsweise die Demenzform, das Demenzstadium, das Alter, die Kultur, die Muttersprache sowie die Biografie der Zielgruppe erfolgen soll. Auch die Unterstützung im einfachen Zugang von Informationen wird benötigt. Den Versand von Unterlagen zur Vorbereitung wünschen sich Menschen mit Demenz und Angehörige mehrere Tage vor dem geplanten Treffen. Personengruppen geben auch die Notwendigkeit des technikgestützten, zielgruppenspezifischen Informationserhalts via Internetseite, App oder Cloudsystem an.

#### II „Beitragen“.

Rückmeldungen der Stakeholder zum Projekt sind ernst zu nehmen. Die zuvor beschriebene Beziehungsbildung und damit korrespondierendes Vertrauen mit dem Projektteam beeinflussen, wie transparent Feedback gegeben wird. Menschen mit Demenz benötigen hierzu präzise formulierte Aufgabenstellungen und eine konkrete Ansprechperson zur Beantwortung offener Fragen.

#### III „Einfluss nehmen“.

In der Gruppe bedarf es einer Rollen- und Aufgabendefinition aller Stakeholder. Daraus leitet sich die Notwendigkeit ab, Klarheit über das Projektverständnis aus diesen Rollen heraus zu erhalten, damit jede Person eigenständig und für sich selbst entscheiden kann, wie sie die Aufgabe des „Einflussnehmens“ in Diskussionen gestalten möchte. In Diskussionen wünschen sich Personengruppen eine angemessene Sprechgeschwindigkeit und eine verständliche Sprache sowie die Wiederholung von Argumenten, die nicht mit der Anwesenheit von Menschen mit Demenz zu begründen ist.

#### IV „Mitgestalten“.

Zielgruppenübergreifend wird eine Unterstützung durch eine feste Ansprechperson in Aufgabenverteilung und Zeitmanagement gewünscht. Stakeholder benötigen Aufgabenzuweisungen, die sich an ihren Ressourcen und Kompetenzen, wie beispielsweise berufliche Expertisen (z. B. Fremdsprachenkompetenzen für Übersetzungsaufgaben) ausrichten. Hinsichtlich des Zeitmanagements bedarf es eines konkreten Zeitplans für die Aufgaben sowie des verbindlichen Klärens zeitlicher Kapazitäten. Im Projektverlauf wünschen sich Stakeholder, zur Projektentwicklung durch Quartalsberichte und halbjährliche Feedbackrunden informiert zu werden.

Menschen mit Demenz sowie Angehörige benötigen eine:n wissenschaftliche:n Mitarbeiter:in als Begleitperson, Ansprechpartner:in und Forschungskolleg:in, mit der sie zusammen Aufgaben durchführen und offene Fragen klären. Menschen mit Demenz und Angehörige benötigen in Seminaren Unterstützung in der Technikkompetenz. Angehörige benötigen Unterstützung im Wissenserwerb durch Workshops und Literaturempfehlungen zu wissenschaftlichem Arbeiten. Menschen mit Demenz und Angehörige benötigen darüber hinaus Unterstützung im Alltag, beispielsweise bei dem Transport zum Veranstaltungsort.

#### V „Mitforschende sein“.

Zielgruppenübergreifend beinhaltet die Unterstützung die Anforderung einer Vereinbarung zur Zusammenarbeit. Es wird eine Moderation gefordert, die sowohl als Ansprechperson als auch für die Koordination des Projektes zuständig ist und einen regelmäßigen Projektaustausch insbesondere in Präsenz terminiert. Um Mitforschende sein zu können, muss ein Angebot zur Wissensvermittlung gegeben werden. Da sich Menschen mit Demenz in der Eigenverantwortung des Vorträgehaltens sehen, fordern sie Unterstützung im Einüben in der Gewissheit, dass sie im Krankheitsfall vertreten werden.

## Diskussion

Unser vorläufiges Framework zeichnet sich im Vergleich zu anderen Untersuchungen und Empfehlungen zum Einbezug von Menschen mit Demenz und ihren Angehörigen dadurch aus, dass in 5 verschiedenen Ausprägungen der Zusammenarbeit Voraussetzungen, Aufgabenfelder und Unterstützungsmaßnahmen differenziert herausgearbeitet wurden. Die Inhalte stützen dennoch die Ergebnisse und daraus abgeleiteten Empfehlungen anderer Studien. Die Voraussetzung der Beziehungsgestaltung, des Vertrauensaufbaus und vorhandener Sozialkompetenzen, die Menschen mit Demenz und Angehörige während der gemeinsamen Gespräche wiederkehrend benannten, stützt die Empfehlung von Griffith et al. [7]. Sie betonen, dass die Entwicklung von Beziehungen im Verlauf des Forschungsprozesses in Form von wiederkehrenden und flexiblen Treffen gegenseitigen Respekt hervorruft. Gove et al. [5] betonen neben der kontinuierlichen Beachtung des emotionalen Wohlergehens der Menschen mit Demenz insbesondere die Reziprozität in Form sich ergänzender Kompetenzen und Beiträge von Forschenden und Menschen mit Demenz als Aspekt, der Beziehungen fördert und gegenseitigen Respekt hervorruft.

Die Formen der Zusammenarbeit in den 5 Ausprägungen der Einbindung, die Personengruppen in den Treffen benannten, machen deutlich, dass Menschen mit Demenz, Angehörige sowie Expert:innen aus der akut- und langzeitstationären Pflege in alle Stufen des Forschungsprozesses aktiv eingebunden werden möchten. Die Relevanz der kontinuierlichen Einbindung im fortlaufenden Forschungsprozess wird auch in der Literatur deutlich [5, 7, 15]. Hier wird insbesondere die Planung einer Studie im Kontext der Berücksichtigung von Personengruppen in der Antragstellung und Budgetplanung unterstrichen [5]. Dennoch betonen Gove et al. [5], dass Forschende die finanzielle und administrative Gesamtverantwortung zu tragen haben. Die durch die Stakeholder als notwendig beschriebene Unterstützung zu gemeinsamem Forschen korrespondiert mit den Forderungen der Alzheimer Europe [5]. Dabei ist es von besonderer Relevanz, dass Barrieren, die durch die Krankheitsspezifika der Personen mit Demenz einhergehen, beachtet und identifiziert werden, sowie die freie und autonome Entscheidungsfähigkeit der Person mit Demenz im Kontext partizipativer Forschung adressiert werden [3].

## Limitationen

Das Modell von Finderup et al. [4] wurde nicht für und auch nicht mit Menschen mit Demenz entwickelt. Die in dieser Studie übersetzte Version wurde jedoch mit Menschen mit Demenz, Angehörigen sowie Expert:innen diskutiert und reflektiert. Auf diese Weise hatten die Stakeholder sowohl Einfluss auf die Gestaltung als auch die Inhalte der partizipativen Vorgehensweise.

Die teilnehmenden Stakeholder hatten bereits Erfahrungen in der Teilnahme an Forschungsprojekten gesammelt und konnten somit ihre Expertise zu Formen der Zusammenarbeit und benötigter Unterstützung einbringen. Die Ergebnisse unserer Studie sind nicht repräsentativ und nicht übertragbar auf jene Stakeholder wie auch Menschen mit Demenz, die über gar keine bis wenig Forschungserfahrung verfügen. Darüber hinaus konnten aus Gründen fehlender zeitlicher Ressourcen nur 2 Personen mit Demenz teilnehmen. Aus diesem Grund sind die Ergebnisse nicht repräsentativ für diese Personengruppe. Um in der Wissenschaft sozialer Ungleichheit entgegenzuwirken [1], sind Studien notwendig, um ihre Perspektiven aktiv zu berücksichtigen und einzubeziehen, mit dem Ziel, das vorläufige Framework des gemeinsamen Forschens weiterzuentwickeln.

Das vorläufige Framework für gemeinsames Forschen bietet ein erstes allgemeines Gerüst, das Forschenden in zukünftigen Projekten zu partizipativer Forschung in Deutschland als Orientierung dient. In diesem Zusammenhang findet das Framework bereits Anwendung in dem Projekt „DECIDE-SR“ mit dem Ziel, gemeinsam mit Stakeholdern ein systematisches Review zu planen und umzusetzen [16, 17]. Mit voranschreitender Nutzung und den wachsenden Erfahrungen in Projekten soll das vorläufige Framework zu einem Framework des gemeinsamen Forschens verdichtet werden.

## Fazit für die Praxis

Menschen mit Demenz und ihre Angehörige möchten aktiv in die Versorgungsforschung einbezogen werden und können konkrete Vorstellungen zu Tätigkeitsbereichen in den verschiedenen Ausprägungen der Einbindung beschreiben. Gleichzeitig benennen sie konkrete Aspekte, welche Unterstützung sie in welcher Ausprägung der Einbindung in Forschungsprozessen benötigen. Forschende sollten in der Zusammenarbeit mit diesen Personengruppen gezielt auf diese Unterstützungsbedarfe eingehen.

## Supplementary Information

Das Zusatzmaterial beinhaltet methodische Erläuterungen zum theoretischen Rahmen, zur Rekrutierung der Stakeholder, zur Ethik sowie nähere Erläuterungen zu den Stakeholdern.

## Data Availability

Der Zugang zu den Daten ist auf Anfrage bei dem Deutschen Zentrum für Neurodegenerative Erkrankungen (DZNE) e. V., Standort Witten, möglich. Bitte kontaktieren Sie das Datenmanagement des Deutschen Zentrums für Neurodegenerative Erkrankungen (DZNE) e. V., Standort Witten (data-management-witten(at)dzne.de).
