# Supplementary material for: Participation in health services research: The development of a preliminary framework for joint research involving people living with dementia, relatives and various professional groups
Source: Z Gerontol Geriatr. 2025 Sep 6;59(5):399–405. [Article in German] doi: 10.1007/s00391-025-02494-6 (PMC13433721; doi:10.1007/s00391-025-02494-6)
Supplement: Supplementary file 1 [file 391_2025_2494_MOESM1_ESM.docx]

# **Supplementary Material**

## Theoretischer Rahmen

Das Modell von Finderup et al. [1] wurde als theoretischer Rahmen genutzt, da es insbesondere Forschende bei der Entscheidung zu Ebenen und Methoden partizipativer Forschung unterstützt. Dieses Modell basiert auf dem Canada Public Involvement Continuum [3] und wurde von elf Forschenden unterschiedlicher beruflicher Ebenen im Rahmen von Workshops entwickelt und anschließend auf Benutzerfreundlichkeit getestet [1]. Es soll neuen Forschenden einen Überblick geben, wie Personengruppen (in fünf Ausprägungen der Einbindung) in die Forschung eingebunden werden können. Dabei variiert die Einbindung auf einem Kontinuum von passiver Teilnahme hin zum aktiven Mitwirken: 1) Inform and Educate, 2) Gather Information, 3) Discuss, 4) Engage, und 5) Partner. Die Ausprägungen wurden im Projektteam in die deutsche Sprache übersetzt und an den deutschsprachigen Kontext adaptiert.

### Rekrutierung der Stakeholder

Für die Rekrutierung von Stakeholdern wurden Informationsflyer angefertigt. Um ein vorläufiges Framework für gemeinsames Forschen zwischen Menschen mit Demenz, Angehörigen sowie Mitarbeitenden einer Forschungsinstitution und Expert:innen aus der langzeitstationären Altenhilfe und dem akutstationären Setting zu erstellen, erfolgte die Kontaktaufnahme an ein Netzwerk von Stakeholdern, zu denen bereits eine mittel- bis längerfristige Zusammenarbeit am Deutschen Zentrum für Neurodegenerative Erkrankungen (DZNE) Standort Witten besteht. Demnach konnte sichergestellt werden, dass potenzielle Teilnehmende bereits Expertise mit Forschungsprojekten gesammelt haben und auf Basis des übersetzten Modells von Finderup et al. [1] Aussagen dazu geben können, wie sie sich eine partnerschaftliche Forschung zum Thema „Versorgung von Menschen mit Demenz“ vorstellen können. Aus diesem Netzwerk wurden Menschen mit Demenz, Angehörige sowie Expert:innen aus dem langzeitstationären und akutstationären Setting über das Vorgehen informiert und nach ihrer Bereitschaft gefragt, an dem Vorgehen mitzuwirken. Stimmten sie zu, wurde ein gemeinsames virtuelles Meeting vereinbart. Da das Projekt eine Laufzeit von zwei Monaten hatte, wurden über das Netzwerk hinaus keine weiteren Stakeholder für das Projekt angefragt.

### Stakeholder/Sample

Aufgrund der unterschiedlichen Personengruppen wurden die Meetings und die dazugehörigen Präsentationsfolien einheitlich in einfacher Sprache vorbereitet. Mit Menschen mit Demenz wurden zwei weitere Meetings durchgeführt, da der zeitliche Umfang des ersten virtuellen Meetings für die gemeinsame Diskussion nicht ausreichte.

Auf Seiten der Forschenden des Deutschen Zentrum für Neurodegenerative Erkrankungen (DZNE) Standort Witten haben insgesamt drei wissenschaftliche Mitarbeiterinnen der Pflegewissenschaft (ALH, CM, MRM) und eine Projektleitung (MR) mitgewirkt.

### Ethik

Für das partizipative Vorhaben ist kein Ethikantrag notwendig. Dies steht im Einklang mit den Empfehlungen der Alzheimer Europe [2]. Das Projektteam arbeitete mit Stakeholdern unterschiedlicher Personengruppen partizipativ zusammen, die als Mitforschende und nicht als Studienteilnehmende anzusehen sind. Gesprächsinhalte wurden nicht mit einem Tonband aufgezeichnet, sondern mittels Ergebnisprotokoll festgehalten. Daraus folgt, dass der Umfang der Inhaltsanalyse begrenzt war und keine Datensättigung erwartbar war.

Die Gruppe der Teilnehmenden repräsentiert ein heterogenes Sample. Wir vermuten jedoch, dass Gespräche mit Menschen mit Demenz mit unterschiedlichen Formen und Stadien der Erkrankung sowie Expert:innen aus der ambulanten Pflege Hinweise auf zusätzliche Voraussetzungen, Formen der Zusammenarbeit aber auch andere Unterstützungsbedarfe verdeutlicht hätten. Daraus leitet sich die Notwendigkeit der Weiterentwicklung dieses vorläufigen Frameworks ab.

### Datenanalyse

Die Protokolle und Fotos der Metaplantafeln wurden in MAXQDA (VERBI Software, Berlin) [5] überführt und mittels inhaltlich strukturierender qualitativen Inhaltsanalyse nach Kuckartz und Rädiker [4] deduktiv induktiv ausgewertet. Hierzu wurden in einem ersten Schritt folgende deduktive Kategorien aus den Fragestellungen heraus entwickelt: Ausprägungen der Zusammenarbeit und Unterstützungsbedarfe. In einem zweiten Schritt wurden induktiv Subkategorien zu Ressourcen, Arten der Zusammenarbeit und Formen der Unterstützung gebildet. Alle deduktiven und induktiven Kategorien wurden auf das gesamte Material angewandt.

**Referenzen**

1.	Finderup J, Buur LE, Tscherning SC et al. (2022) Developing and testing guidance to support researchers engaging patient partners in health-related research. Res Involv Engagem 8:43

2.	Gove D, Diaz-Ponce A, Georges J et al. (2018) Alzheimer Europe's position on involving people with dementia in research through PPI (patient and public involvement). Aging Ment Health 22:723-729

3.	Government of Canada (2000) The Health Canada Policy Toolkit for Public Involvement in Decision Making. https://www.canada.ca/content/dam/hc-sc/migration/hc-sc/ahc-asc/alt_formats/pacrb-dgapcr/pdf/public-consult/2000decision-eng.pdf

4.	Kuckartz U, Rädiker S (2024) Qualitative Inhaltsanalyse. Methoden, Praxis, Umsetzung mit Software und künstlicher Intelligenz. Beltz Juventa, Weinheim Basel

5.	Verbi Software (2021) MAXQDA 2022 [computer software]. In:VERBI Software, Berlin, Germany
